# Unique genotypic features of HIV-1 C gp41 membrane proximal external region variants during pregnancy relate to mother-to-child transmission via breastfeeding

**DOI:** 10.46439/pediatrics.1.003

**Published:** 2021

**Authors:** Li Yin, Kai-Fen Chang, Kyle J. Nakamura, Louise Kuhn, Grace M. Aldrovandi, Maureen M. Goodenow

**Affiliations:** 1 Molecular HIV Host Interaction Section, National Institute of Allergy and Infectious Diseases, National Institute of Health, Bethesda, MD, USA; 2 Illumina Inc., San Diego, CA, USA; 3 Gertrude H. Sergievsky Center, College of Physicians and Surgeons, and Department of Epidemiology, Mailman School of Public Health, Columbia University, New York, NY, USA; 4 Department of Pediatrics, Sabin Research Institute, Children’s Hospital Los Angeles, Los Angeles, CA, USA

**Keywords:** Next generation sequencing, Subtype C, HIV-1 gp41 MPER, MTCT, Breastfeeding, Biodiversity, Hydropathy, Charge

## Abstract

Mother-to-child transmission (MTCT) through breastfeeding remains a major source of pediatric HIV-1 infection worldwide. To characterize plasma HIV-1 subtype C populations from infected mothers during pregnancy that related to subsequent breast milk transmission, an exploratory study was designed to apply next generation sequencing and a custom bioinformatics pipeline for HIV-1 gp41 extending from heptad repeat region 2 (HR2) through the membrane proximal external region (MPER) and the membrane spanning domain (MSD). MPER harbors linear and highly conserved epitopes that repeatedly elicits HIV-1 neutralizing antibodies with exceptional breadth. Viral populations during pregnancy from women who transmitted by breastfeeding, compared to those who did not, displayed greater biodiversity, more frequent amino acid polymorphisms, lower hydropathy index and greater positive charge. Viral characteristics were restricted to MPER, failed to extend into flanking HR2 or MSD regions, and were unrelated to predicted neutralization resistance. Findings provide novel parameters to evaluate an association between maternal MPER variants present during gestation and lactogenesis with subsequent transmission outcomes by breastfeeding.

**Importance:**

HIV-1 transmission through breastfeeding accounts for 39% of MTCT and continues as a major route of pediatric infection in developing countries where access to interventions for interrupting transmission is limited. Identifying women who are likely to transmit HIV-1 during breastfeeding would focus therapies, such as broad neutralizing HIV monoclonal antibodies (bn-HIV-Abs), during the breastfeeding period to reduce MTCT. Findings from our pilot study identify novel characteristics of gestational viral MPER quasispecies related to transmission outcomes and raise the possibility for predicting MTCT by breastfeeding based on identifying mothers with high-risk viral populations.

## Introduction

Mother-to-child HIV-1 transmission (MTCT) can occur during pregnancy, delivery (perinatally) or breastfeeding and contributes substantially to global morbidity and mortality for children under-5 years of age. Rates of perinatal MTCT range from 15% to 45% in the absence of any interventions but can be reduced to less than 5% with appropriate antiretroviral treatment [1–5]. HIV-1 transmission through breastfeeding accounts for 39% of MTCT, and continues to be a major route of pediatric infection in developing countries [6], where access to interventions for interrupting transmission is limited [7].

Viruses that establish MTCT either perinatally or through breastfeeding display limited diversity, as well as relatively short and under-glycosylated gp120 regions [8–12], similar to gp120 regions among transmitter/founder viruses in general [13–16]. The membrane-proximal external region (MPER) of gp41 contains linear epitopes for broadly HIV-1 neutralizing antibodies (bn-HIV-Abs), including 2F5, 4E10, 10E8, Z13e1, and most recently LN01, DH511, VRC42 and PGZL1, and is accessible to plasma bn-HIV-Abs [17–26]. MPER targeting bn-HIV-Abs show outstanding breadth by neutralizing over 90% of viral strains on multiclade panels [19,22,24,26,27]. Although MPER targeting bn-HIV-Abs arose independently from different individuals infected by various clades [19,22,24,26,28], their epitopes overlap extensively, suggesting epitope conservation, immunogenesis, and antibody accessibility and supporting vaccine efforts [29–32]. Elevated maternal antibody titers to HIV-1 envelope (*env*) gp41 and/or gp120 epitopes are directly associated with perinatal MTCT [33–37]. Our previous study of HIV-1 MPER sequences from HIV-1 infected mother-baby pairs in the Zambia Exclusive Breastfeeding Study (ZEBS), a clinical trial to prevent MTCT of HIV-1 through breast milk [38–40], suggests that polymorphisms in MPER occur naturally and can confer resistance to broadly neutralizing anti-MPER antibodies [40]. Thus, it is plausible to hypothesize that HIV-1 MPER variants in mothers who transmit HIV-1 to their babies by breastfeeding (TM) display a greater extent of genetic polymorphism in MPER compared to those who do not transmit (NTM).

Cross-sectional as well as longitudinal studies of cell-free HIV-1 find persistent mixing and synchronous evolution of viruses between plasma and breast milk in the ZEBS and other cohorts indicating that HIV-1 quasispecies in plasma are representative of virus populations in breast milk [38,41–45], although compartmentalization of cell-associated viruses in breast milk is reported in other studies [41,46]. A sophisticated phylogenetic analysis of longitudinal HIV-1 *env* V1-V5 sequences from plasma and breast milk of transmitting mothers suggests that the most common ancestral virus(es) in breast milk originate during the second or third trimester of pregnancy, close to the onset of lactogenesis [38]. Consequently, plasma HIV-1 variants during pregnancy might harbor genetic features related to subsequent breast milk transmission.

To examine the relationship between maternal viruses during gestation and subsequent transmission outcomes through breastfeeding, a pilot study of ZEBS maternal plasma subtype C HIV-1 from second or third trimester of pregnancy were evaluated by next generation sequencing (NGS) to provide broad coverage of HIV-1 quasispecies at the population level and sensitive detection of low-frequency variants. A custom bioinformatic pipeline was developed to assess biodiversity, amino acid substitutions within linear epitopes of known bn-HIV-Abs targeting gp41 MPER, and biochemical features (hydropathy and charge) of plasma subtype C HIV-1 gp41 MPER variants and compared to the adjacent heptad repeat region 2 (HR2) or membrane spanning domain (MSD) among mothers who transmitted or did not transmit HIV-1 through breastfeeding.

## Materials and Methods

### Study cohort

A nested, case-control study included a subset of eight women infected by subtype C HIV-1 enrolled in ZEBS [38–40]. All subjects were therapy-naive, except for a single peripartum dose of nevirapine according to the Zambian government guidelines during the enrollment period (2001–2004). Written informed consent for participation in the ZEBS study was obtained from all participants. From the larger cohort, our study included plasma samples from four women who transmitted HIV-1 during the early breastfeeding period (TM) (defined by infants who became HIV-1 DNA positive after 42 days following prior negative tests), and four infected women who did not transmit HIV-1 (NTM) [defined by infants who remained HIV-1 DNA negative through the completion of all breastfeeding for a median (quartile range) (QR) of 6.5 (4.0–18.8) months] (Table 1). Maternal plasma samples were collected prospectively during the second/third trimester of pregnancy [median (QR): 80 (32–164) days before delivery] (Table 1). At the time of sampling, the two groups of women were balanced for median (QR) of age [TM, 25.5 (22.5–31.5) years *vs.* NTM, 27.0 (20.3–34.5) years] (p=0.87), CD4 T-cell count [TM, 146 (117–187) cells/µl *vs.* NTM, 202 (132–240) cells/µl] (p=0.27), plasma viral load [TM, log_10_ 5.2 (4.9–5.5) HIV-1 RNA copies/ml plasma *vs.* NTM, log_10_ 5.2 (5.0–5.3) HIV-1 RNA copies/ml plasma] (p=1.00), and breastfeeding period [TM, 4.0 (4.0–11.5) months *vs.* NTM, 6.5 (4.0–18.8) months] (p=0.53). This genetic protocol was approved by the Institutional Review Boards of the University of Florida, the Sabin Research Institute, and Children’s Hospital Los Angeles.

### Generation of amplicon library

Viral RNA was extracted from 280µl of plasma using QIAamp Viral RNA Mini Kit (Qiagen, Valencia, CA). A library of HIV-1 *env* gp41 amplicons [342 nucleotides in length, including pre-HR2 (105 nucleotides) HR2 (102 nucleotides), MPER (66 nucleotides), and 5’ MSD (69 nucleotides)] was generated for each subject from 2,000 HIV-1 RNA copies by RT-PCR using SuperScript™ One-Step RT-PCR (Invitrogen, Carlsbad, California) followed by amplification using GoTaq colorless Master Mix (Promega, Madison, WI) [47]. First round amplification used forward primer 251 (5’-GGG GCT GCT CTG GAA AAC TCA TCT-3’) and reverse primer 585 (5’-AAT GGT GAG TAT CCC TGC CTA ACT-3’) (nucleotide positions 8,011–8,034 and 8,345–8,368, respectively, in HIV-1_HXB2_ genome [48]; 7,387–7,410 and 7,721–7,744, respectively in HIV-1_ETH2220_ genome), while second round amplification used forward A-257 (5’-CGTATCGCCTCCCTCGCGCCATCAG GCT CTG GAA AAC TCA TCT GCA CCA-3’) and reverse B-575 (5’-CTATGCGCCTTGCCAGCCCGCTCAG ATC CCT GCC TAA CTC TAT TCA CTA-3’) (nucleotide positions 8,017–8,040 and 8,335–8,358, respectively, in HIV-1_HXB2_ genome; 7,393–7,416 and 7,711–7,734, respectively, in HIV-1_ETH2220_ genome) with adaptors A or B (underlined nucleotides in respective primer) incorporated at the 5’ ends. Amplicons were gel purified using QIAquick Gel Extraction Kit (Qiagen) as described [49], and submitted to the Interdisciplinary Center for Biotechnology Research at University of Florida for Titanium Amplicon 454-pyrosequencing reading from adaptor B using a Genome Sequencer FLX (454 Life Sciences) according to the manufacturer’s protocol.

### Sequence analysis

A bioinformatics pipeline was developed to facilitate analysis of large numbers of HIV-1 gp41 HR2-MPER-MSD sequence reads. The median (QR) number of raw reads was 56,647 (43,142–75,450) per subject. Sequences were submitted to NCBI public access database with accession numbers pending. A quality control step filtered a median (QR) of 7.5% (5.2%−13.2%) low quality reads with ambiguous nucleotides, more than one error in either primer tag, or a length outside mean ± 2 SD length range, leaving median (QR) of 52,408 (37,541–71,533) quality sequences per sample. Depth of sequencing provided median (QR) of 27 (19–36)-fold coverage of input 2,000 HIV-1 RNA copies with no significant difference in sequence number or fold coverage among the samples between the groups. Quality MPER sequences were extracted from the entire HR2-MPER-MSD sequences by aligning to HIV-1_HXB2_ and to HIV-1 subtype C consensus sequence generated from HIV sequence database [50].

Nucleotide sequences were clustered at 3% genetic distance using ESPRIT [49,51,52] to develop a consensus sequence for each cluster that represents a sequence variant. Complexity of the HIV-1 population within each individual was evaluated by neighbor-joining (NJ) phylogenetic tree generated from consensus sequences with the maximum-likelihood composite model implemented in MEGA v5.2 [53,54]. Statistical support was assessed by 1,000 bootstrap replicates. NJ trees were annotated manually in Adobe Illustrator CS4 (Adobe Systems Incorporated, San Jose, CA) to display frequencies of HIV-1 cluster variants. Frequencies of amino acid differences at each position compared to subtype B HIV-1_HXB2_ were calculated. Non-synonymous substitutions resulting in alteration of viral sensitivity to bn-HIV-Abs, including 2F5, 4E10, LN01, DH511, VRC42, PGZL1, 10E8 and Z13e1, were identified by mapping to known resistant/sensitizing mutations (Figure S1) [19,22,24,26,28,40,55–71]. Number and frequency of amino acid differences were compared between TM and NTM sequences. Positive selection at epitope-composing positions was inferred by Phylogenetic Analysis by Maximum Likelihood (PAML) [72]. Hydropathy index and charge of each MPER consensus sequence were calculated using an in-house code [52,73,74].

Polymorphisms across all sequences were evaluated by biodiversity, expressed as operational taxonomic units (OTU), using rarefaction, while Chao1 algorithms in ESPRIT [51]. Rarefaction curves display HIV-1 diversity over sequencing depth, and Chao1 infers maximum biodiversity within 2,000 input HIV-1 RNA copies [49,51,52].

### Statistical analysis

Groups were compared by unpaired t test. Statistical analyses were performed using SAS version 9.1 (SAS Institute, Cary, NC) with P <0.05 (two sided) defined as significant. Logistic regression was used to examine the effects of predicted hydropathy or charge of HIV-1 gp41 MPER and their interactions (exposures) on transmission (outcome).

## Results

### Population structure

**Figure 1:**
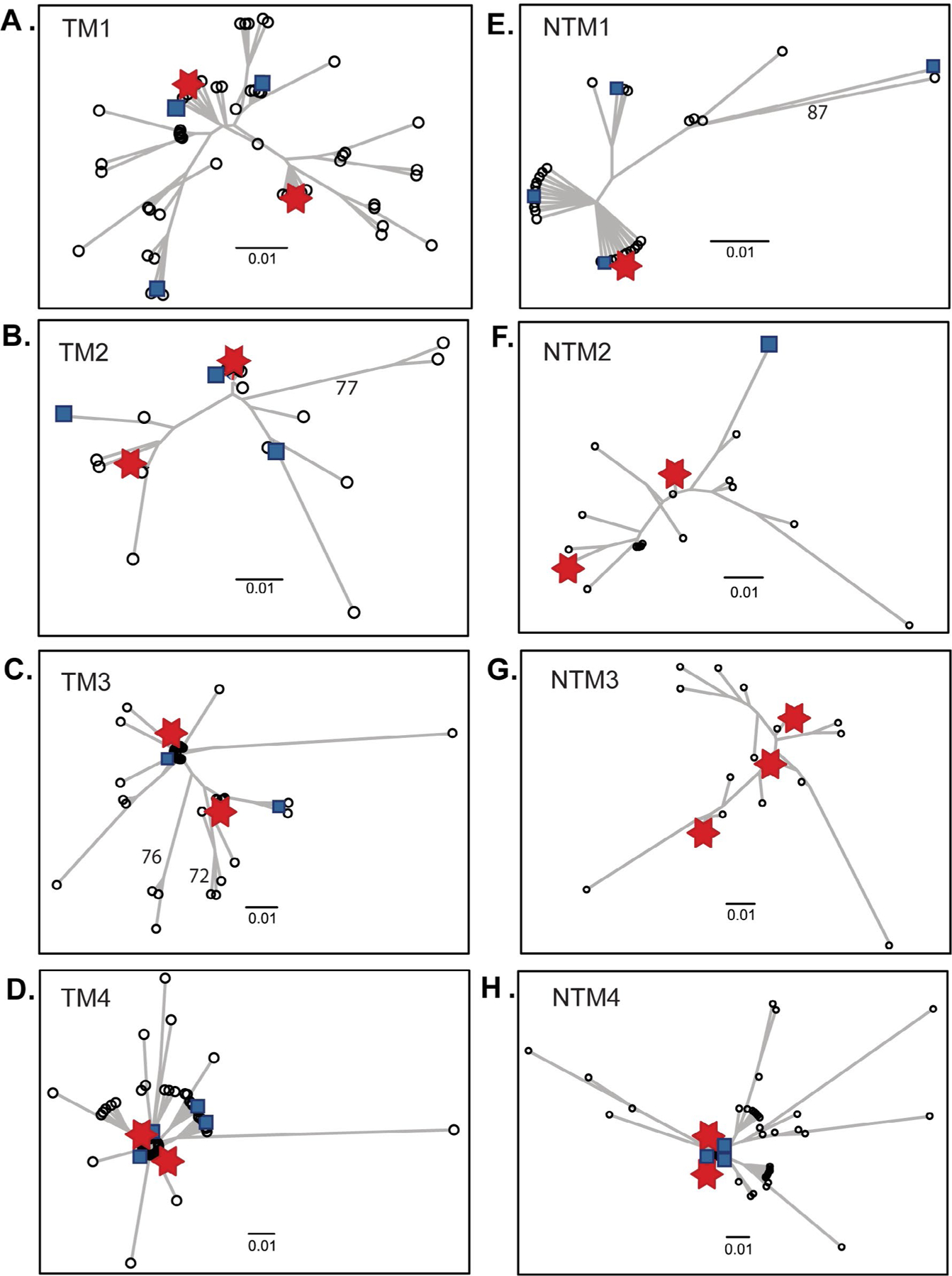
Organization of HIV-1 gp41 MPER populations. An unrooted neighbor-joining tree for each individual was developed from the deep sequencing data set clustered at 3% genetic distance. Each branch represents a consensus sequence of HIV-1 gp41 MPER within 3% genetic distance. Symbols represent the proportion of total deep sequences in a cluster: Ο, ≤ 0.25%; ■, > 0.25 % to 10%; 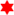 , >10%.

To evaluate the complexity of viral population structure within each individual, unrooted phylogenetic tree were constructed from maternal consensus MPER sequence clusters. Overall, the analysis showed that sequences were correctly assigned to each individual with no sequence mixing among subjects. Within each subject HIV-1 population were organized into one to three dominant clusters with thousands of sequences per cluster (Figure 1). Dominant sequence clusters generally included a median (QR) of 47% (19%−63%) of sequences. Sequences representing 0.25% to 10% of the viral population within an individual also appeared in low frequency (0 to 4) clusters surrounded by swarms of clusters with less abundant variants, usually representing <0.25% of the population. The structure of viral populations based on gp41 regions was indistinguishable between TM and NTM and similar to HIV-1 populations based on gp120 V3 [49].

### Biodiversity of HIV-1 MPER quasispecies

**Figure 2:**
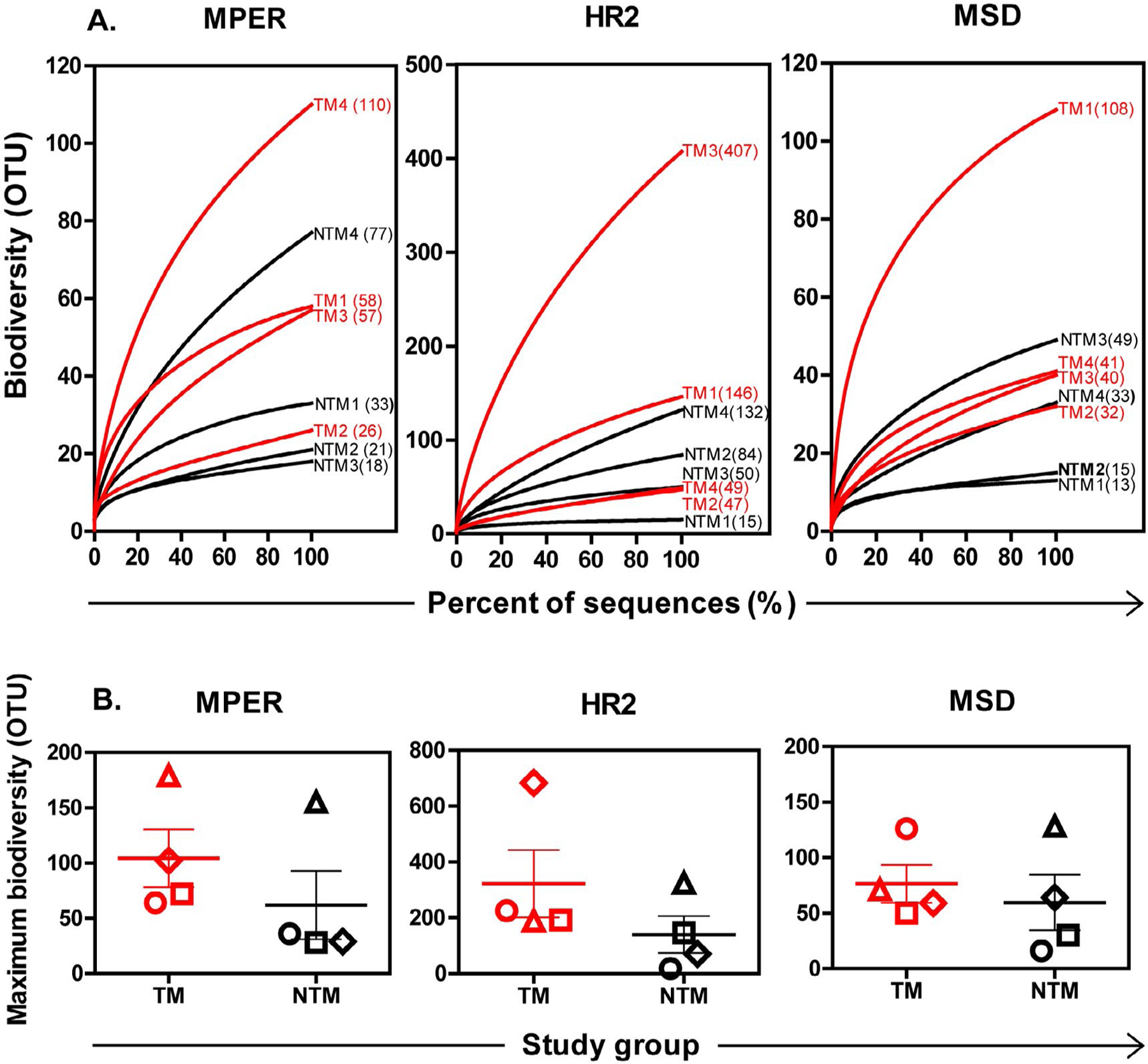
Biodiversity among HIV-1 viral populations. Nucleotide deep sequences of HIV-1 MPER (66 bp), or HR2 (102 bp), or MSD (69 bp) from each individual were clustered at 3% genetic distances and displayed as rarefaction curves (A) and Chao1 values (B). A. Y-axis, number of OTU (number of sequence clusters); x-axis, percent of total deep sequences (sequences sampled ÷ total number of sequences x 100%). Rarefaction curves show HIV-1 variants from TMs (red) or NTMs (black), respectively. Numbers of OTU at the end of curves represent biodiversity calculated from rarefaction curve at the sequence depth (100% of deep sequences). B. Y-axis, maximum number of OTU within 2,000 input viral copies estimated by Chao1 algorithm based on rarefaction curve of HIV-1 variants from each subject [51]; x-axis, study group, TM or NTM, respectively. Symbols: Ο, subject #1; □, subject #2; ◊, subject #3; ∆, subject #4. Red symbols, TM; black symbols, NTM.

Biodiversity of HIV-1 MPER nucleotide sequences within each individual were assessed using rarefaction curves. HIV-1 MPER nucleotide sequences among TM displayed biodiversity ranging from 26 to 110 OTU, which was approximately 50% greater than biodiversity ranging from 18 to 77 OTU among NTM (Figure 2A). When maximum biodiversity within 2,000 HIV-1 RNA copies was estimated, viral populations among TM, compared to populations among NTM, displayed a trend toward greater biodiversity [median (QR): 87 (66–160) OTU versus 33 (28–125) OTU, p=0.33] (Figure 2B).

To determine if differences in biodiversity between TM and NTM were restricted to MPER or extended to adjacent regions in gp41, similar analyses were applied to HR2 and to MSD sequences (Figure 2). Overall, mean estimated maximum biodiversity was more than 2-fold greater in HR2 than in MPER among TM or NTM groups, reflecting in part that the HR2 region (102 nucleotides) is almost twice as long as MPER (66 nucleotides). MSD encoding regions (69 nucleotides) are similar to MPER in length and displayed similar biodiversity between NTM and TM groups, although maximum biodiversity in MSD compared to MPER was reduced among TM group (Figure 2B).

### Amino acid substitutions in HIV-1 MPER

**Figure 3:**
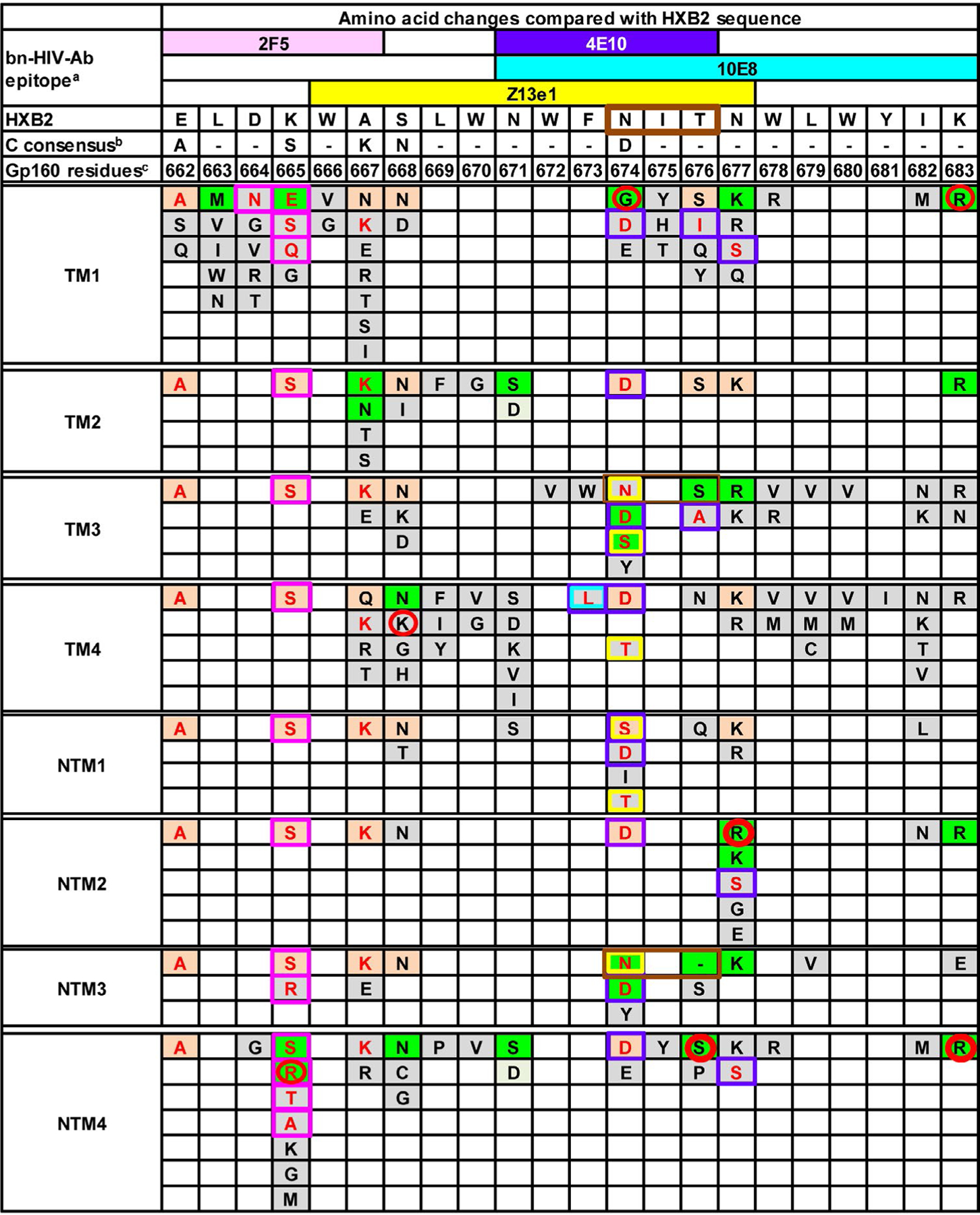
Amino acid changes in MPER compared with HXB2 sequence. Amino acid residue (a single letter code) which differs from HXB2 sequence was shown in each space with red letter representing amino acid residue resistant to bn-HIV-Ab(s) and black letter depicting amino acid with unknown effect on bn-HIV-Ab susceptibility. The K665A labeled by an * is resistant to 2F5 but increasing the sensitivity to 4E10 and PGZL1. Color scheme is used to define frequency of amino acid substitution with beige representing residues in >80% of HIV-1 MPER variants; green depicting residues in >10% to 80% of HIV-1 MPER variants; and grey representing residues in <1% to 10% of HIV-1 MPER variants. Substitutions outlined in pink are resistant to 2F5; purple are resistant to 4E10; dark green are resistant to LN01; orange are resistant to DH511; light green are resistant to VRC42; dark brown are resistant to PGZL1; cyan are resistant to 10E8; and yellow are resistant to Z13e1. Residues under positive selection are circled by red. N-linked glycosylation motifs (NXS/T) are outlined by dark grey. a: epitope reported in HIV molecular immunology database [102] or published articles [19,22,24,26]; b: subtype C consensus sequence generated from HIV sequence database [50]; c: gp160 amino acid residues 662 to 683 are residues 151 to 172 in gp41 [48]; dash (−): amino acid identity between HIV-1_HXB2_ and subtype C consensus.

Biodiversity evaluated at the nucleotide sequence level was reflected in diversity among amino acid residues in MPER (Figure 3), as well as in HR2 and in MSD regions (Figures S2 and S3), indicating that a preponderance of nucleotide polymorphisms within each region involved nonsynonymous changes. HIV-1 MPER variants among TM had changes at more amino acid positions than NTM [median (QR) 14 (12–16) *vs.* 9 (8–14) positions per person, respectively], with amino acid changes in six positions (663, 666, 672, 673, 680 and 681) observed exclusively in TM viral populations. HIV-1 MPER variants from TM also had more amino acid substitutions per position than NTM [median (QR): 7 (4–9) *vs.* 3 (1–7) respectively, p=0.04]. While the MPER reference sequence for subtype B includes a single N-linked glycosylation motif (positions 674 to 676), the subtype C consensus MPER sequence lacks a similar motif. Although some polymorphisms at position 674 would introduce a motif at low frequency, the number of N-linked glycosylation motifs in MPER was similar among viral populations from TM and NTM. MPER amino acid residues under positive selection were limited (N674G and K683R in TM1, S668K in TM4, N677R in NTM2, and K665R, T676S and K683R in NTM4) with no significant difference between TM and NTM (Figure 3).

### Changes in antibody response epitopes in MPER

Amino acid substitutions in MPER epitopes might alter susceptibility (i.e., sensitivity or resistance) to bn-HIV-Abs, including 2F5, 4E10, LN01, DH511, VRC42, PGZL1,10E8 and Z13e1 (Figure S1). A bioinformatics approach was applied to evaluate a potential impact of amino acid polymorphisms in MPER on neutralization susceptibility to bn-HIV-Abs. Overall, the neutralization effects by many of the MPER polymorphisms identified by deep sequencing were undefined (Figure 3). Low frequency 4E10- and PGZL1-sensitizing mutation, K665A [26], was identified only in NTM4. In contrast, some MPER polymorphisms were predicted to be associated with resistance to neutralization by 2F5, 4E10, LN01, PGZL1, 10E8 or Z13e1 [22,26,28,40,56,58,59,61–65,67,68,70,71,75–77]. For example, all subjects harbored dominant virus populations with known subtype C amino substitutions E662A, K665S and A667K conferring 2F5 resistance [58,63,75–77]. Additional 2F5 resistant polymorphisms D664N and K665E/Q/R/T/A [56,58,59,62,64,65,68] were identified in 3 individuals (TM1, NTM3, and NTM4). At least one of four 4E10 resistant substitutions (F673L, N674D/S, T676A/I, or N677S) [28,40,60,61,64,65,68] was identified in each individual. LN01 resistant mutation N674S [22] was observed only in TM3 and NTM1. In contrast, PGZL1-resistant mutations N674E/S/T [26] and resistance substitutions to 10E8 (F673L and N674E/S/T) [22,26,28] appeared in multiple TM (TM1, TM3 and TM4) and NTM (NTM1 and NTM4), while Z13e1 resistant mutations (D674N/S/T) [67] appeared in TM3, TM4, NTM1 and NTM3. No known DH511- or VRC42-resistant mutations [19,24] were observed in any individuals. Overall, polymorphic substitutions with predicted resistance phenotypes were identified with variable frequency in most individuals independent of transmission outcomes.

### Distinct biochemical characteristics of HIV-1 MPER populations between TM and NTM

**Figure 4:**
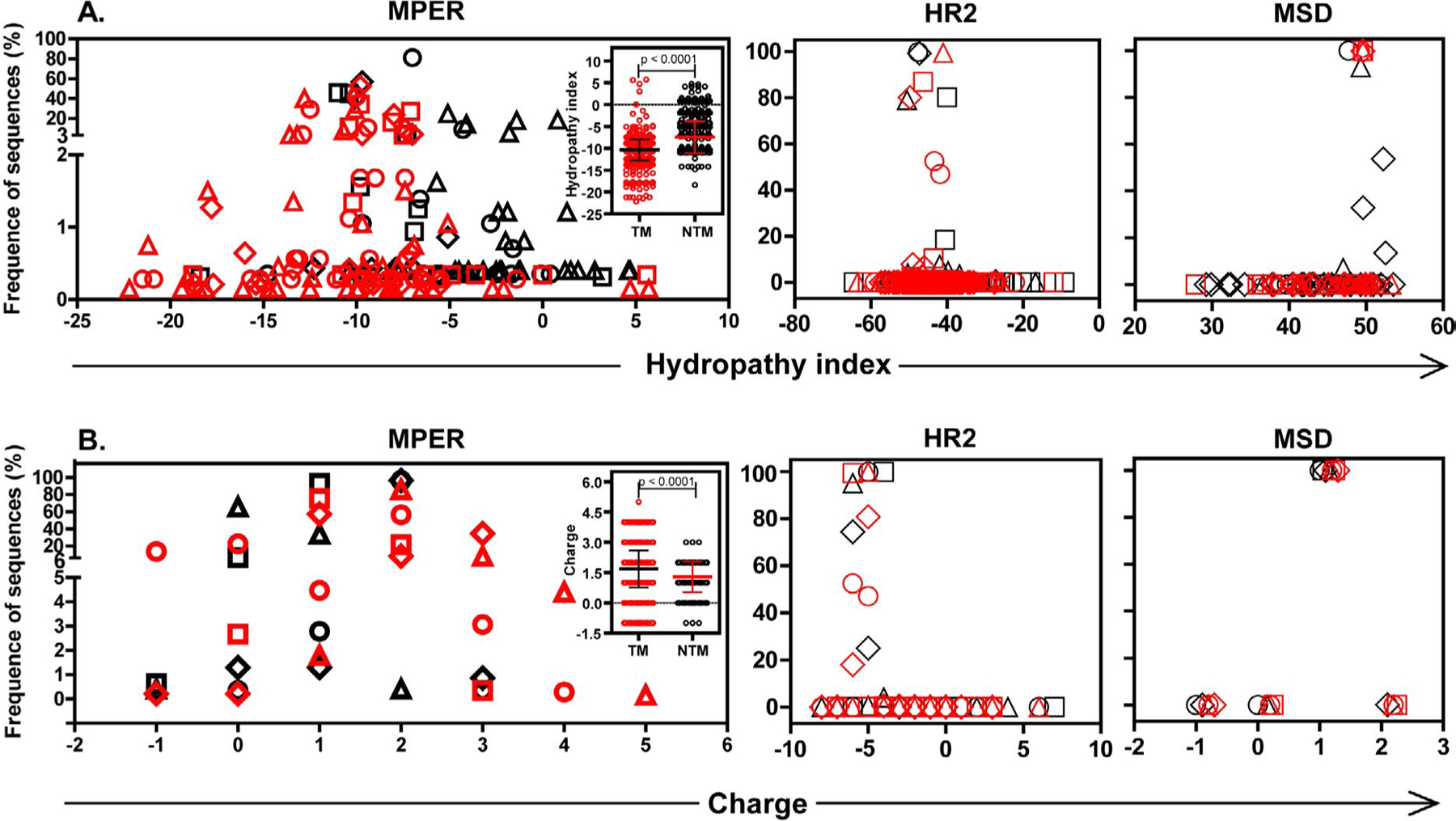
Biochemical characteristics of HIV-1 viral variants. Frequency distribution of A. hydropathy indexes with each symbol representing the percent of consensus sequences with that particular hydropathy index, or B. net charge of HIV-1 viral variants with each symbol depicting percent of consensus sequences with that particular net charge of MPER, HR2 or MSD from TMs (red symbols) or NTMs (black symbols). Symbols: Ο, subject #1; □, subject #2; ◊, subject #3; ∆, subject #4. Inserts in A and B show significantly lower hydropathy index and significantly higher net charge respectively in HIV-1 MPER variants from TM in contrast to NTM with each point representing hydropathy index (A) or net charge (B) of each consensus MPER sequenceeach.

To evaluate if predicted amino acid substitutions might alter the biochemical features of MPER, distribution of hydropathy or charge at the population level within TM or NTM MPER was assessed (Figure 4A). TM viral populations compared with NTM demonstrated a left-shift towards increased frequencies of hydrophilic MPER variants with a median (QR) hydropathy index of −10 (QR, −12.5 to −9.6), significantly lower than NTM variants with a median of −7.3 (QR, −10.4 to −5.1) (p <0.0001). The difference in hydropathy index between TM and NTM was concentrated among variants that appeared with reduced frequency (≤20%) (P <0.0001), but not among high frequency variants (>20%) (p=0.34). Low-frequency variants were uniquely identified by NGS, and not found when clonal or single genome sequences were analyzed [40] (data not shown). When charge of MPER amino acids was assessed, a clear right-shift towards an increase in frequencies of MPER variants with greater positive charges occurred in TM with significantly greater net charges (median 2.0; QR, 1.0 to 2.0) compared with NTM (median 1.0; QR, 1.0 to 2.0) (p <0.001) (Figure 4B). The distinct differences in biochemical features between TM and NTM gp41 populations were restricted to MPER and failed to extend into flanking HR2 or MSD domains (Figure 4).

Logistic regression analysis indicated that an increase in MPER hydrophobicity was significantly associated with reduced odds of transmission by breast feeding (p <0.0001), while positive charged MPER regions showed a close relationship with breast milk transmission (p <0.0001). Logistic regression statistics revealed a significant interactive effect on transmission between hydropathy and charge (p <0.0001). For negative, neutral or positive charged regions, odds ratios were 0.741, 0.416 and 0.781 respectively for a one-unit increase in net hydropathy (95% confidence interval 0.738–0.744, 0.413–0.419 and 0.699–0.873, respectively). Charge has an opposite effect on transmission for negative and positive hydropathy. Increase of net charge was significantly associated with reduced odds of transmission for negative hydropathy (OR=0.627, 95% CI, 0.622–0.632), while for positive hydropathy, net charge increase was significantly associated with elevated odds of transmission (OR=6.358, 95% CI, 3.772–10.718).

## Discussion

Breast milk is essential for infant development and health particularly in resource limited settings [78–81]. Unfortunately, breast feeding remains a major source of global pediatric HIV-1 infection reflecting, in part, limited parameters to identify women at high risk for viral transmission by breastfeeding and the challenges of providing therapeutic interventions for the duration of the breastfeeding period [82–85]. HIV-1 variants that establish new infections by breastfeeding generally occur at low frequency in the transmitting viral population, are characterized by shorter and underglycosylated gp120 Envelopes, and may represent escape from neutralizing antibodies targeting epitopes in both gp120 and gp41 MPER [9–12,86]. Our exploratory studies of HIV-1 variants by metagenomic approaches identified distinct features of gestational MPER populations that distinguished between women who did or did not subsequently transmit HIV-1 during breastfeeding. Transmission outcome groups in our study were well balanced in age, plasma viral load, CD4 T-cell counts and breastfeeding practices, which in combination with the depth of sequencing from each individual provided statistical sensitivity. As anticipated virus populations in plasma during pregnancy among women who subsequently transmitted HIV-1 via breastfeeding displayed greater biodiversity. A higher frequency of HIV-1 MPER variants with hydrophilic and positively charged amino acid residues among TM compared with NTM was discovered. The characteristics could only be evaluated at the population level by NGS, as conventional clonal sequencing biases the population towards dominant variants. Phenotypic differences in peripheral blood viral populations overtime that related to subsequent transmission were evident by the third trimester of pregnancy about the time of lactogenesis [38]. While our current study was designed as a cross sectional comparison of maternal virus populations during gestation, whether or not biochemical differences among maternal viral populations present during pregnancy persist during breastfeeding and are related to infecting cell-free or cell-associated viruses in nursing babies are important questions for subsequent studies [87].

Positive selection for any single amino acid change was limited, as was modulation of glycan motifs across MPER. Sensitivity to bn-HIV-Ab, either alone or in combinations, by the novel amino acids in each MPER allele within an individual is difficult to predict with complete accuracy, may differ by subtype [86] and necessitates direct assessment for neutralization susceptibility [88]. Absence of clear bn-HIV-Ab resistance genotypic profiles during pregnancy that distinguish between TM and NTM does not rule out a subsequent role for neutralization resistance in MTCT by breast milk. Yet, polymorphic amino acid positions within MPER during pregnancy frequently mapped outside motifs associated with known bn-HIV-Ab, raising the possibility that factors other than antibody selection contribute to the differences in MPER characteristics between TM and NTM. For example, a significant role in membrane fusion played by MPER requires functional assays to evaluate the consequences by biochemical variants of MPER for viral entry into different host cells or for crossing mucosal barriers.

HIV-1 gp41 MPER plays a critical role in HIV-1 fusion by perturbing the architecture of the bilayer envelope [89–91]. Distribution of hydrophobic amino acid in MPER can modulate membrane fusion [90,92]. Electrostatic interaction between viral particle and negatively charged lipid membrane may also play a role in viral entry [93]. Antibody-membrane interactions for effective engagement with antigens is introduced as a relatively new concept upon the discovery of anti-MPER antibodies against HIV. Electrostatic and hydrophobic association of antibody to the viral membrane are reported to be essential for efficient epitope binding [94,95]. A study of 2F5 observed that the charge of amino acid residual affects ionic interactions between MPER and 2F5 particularly in core epitopes, while hydrophobic interaction between epitope residuals and/or between antibody and epitope is required for stability of epitope-antibody binding [94]. A recent study by Carravilla P et al. [95] demonstrated that 4E10 binding to virus-like lipid bilayer was disrupted by deletion of the hydrophobic residues or removal of charged lipids, and was enhanced by increasing the overall negative charge. In addition, nonspecific electrostatic antibody-lipid interactions increase 4E10 affinity to Env by providing extra contact sites on the viral surface, enlarging the interacting area, and/or facilitating the insertion of the Ab in the membrane after MPER engagement, thus stabilizing the 4E10-Env complex [95]. The decrease in hydrophobicity and increased in positive charge in MPER in MPER variants from TM mothers in this study may lead to reduced interaction between MPER and MPER targeting antibodies, and thus favored HIV-1 transmission. Logistic regression analysis indicated an interactive effect of hydropathy and charge of HIV-1 MPER variants on breast milk transmission outcome in our study. Similar to our study of gp41 MPER, a significant difference in hydropathy in gp120 between TM and NTM in intrauterine transmission was reported in another study [96], suggesting that intrauterine transmission is associated with maternal envelope quasispecies with altered cellular tropism or affinity for coreceptor molecules expressed on cells localized in the placenta. Together, both studies raise the possibility that antibody-independent mechanisms might contribute to transmission.

A novel aspect of our study is that differences in MPER were compared to flanking regions in gp41. While MPER regions displayed a trend toward increased maximum biodiversity, the striking biochemical characteristics of viral populations associated with MTCT by breastfeeding were restricted to MPER. Although HR2 and MSD segments that flank MPER were diverse, patterns of diversity were unrelated to transmission outcomes, perhaps reflecting HR2 interactions with HR1 or a role for MSD in anchoring gp41 in membranes [97–102]. Overall, deep sequencing coupled with an efficient bioinformatics pipeline provided unprecedented coverage of HIV-1 gp41 MPER quasispecies combined with sensitive detection of low frequency variants that can only be captured by high coverage of input viral copies. Low frequency variants within viral populations are particularly critical and clinically relevant as transmitting viruses. Our proof of principle studies identified months before transmission detailed characteristics of viral quasispecies related to transmission outcomes. By taking into consideration of biodiversity and amino acid polymorphisms increasing antibody resistant or altering the amino acid charges and hydropathies, results raise the possibility for identifying mothers with high-risk viral populations, who might benefit from MPER-targeted bn-HIV-Ab cocktails to reduce transmission during the breastfeeding period.

## Supplementary Material

JCPN-21-003_Supplementary file

## Figures and Tables

**Table 1:** Demographic, immune and viral characteristics of study subjects.

Study group	Maternal					Infant age (days)	
	Age (years)	Trimester	CD4 T cell (cells/µl)	Viral Load^a^	Duration of breastfeeding (months)	Negative PCR^b^	Positive PCR^b^
**TM1**	22	2^nd^	154	5.3	14	34	62
**TM2**	27	3^rd^	110	5.5	4	35	63
**TM3**	33	3^rd^	198	5.0	4	37	70
**TM4**	24	3^rd^	137	4.9	4	28	63
**NTM1**	19	3^rd^	181	5.3	4	738	
**NTM2**	24	2^nd^	115	5.0	22	731	
**NTM3**	36	3^rd^	223	5.3	4	730	
**NTM4**	30	2^nd^	246	5.1	9	639	

a Log10 HIV-1 RNA copies/ml plasma;

b HIV-1 DNA
