# Supplementary figures and images for: Unique genotypic features of HIV-1 C gp41 membrane proximal external region variants during pregnancy relate to mother-to-child transmission via breastfeeding
Source: J Clin Pediatr Neonatol. Author manuscript; Available in PMC 2021 Sep 21. (PMC8454918; doi:10.46439/pediatrics.1.003)

**Fig. S1.**

[illegible]

Fig. S2.

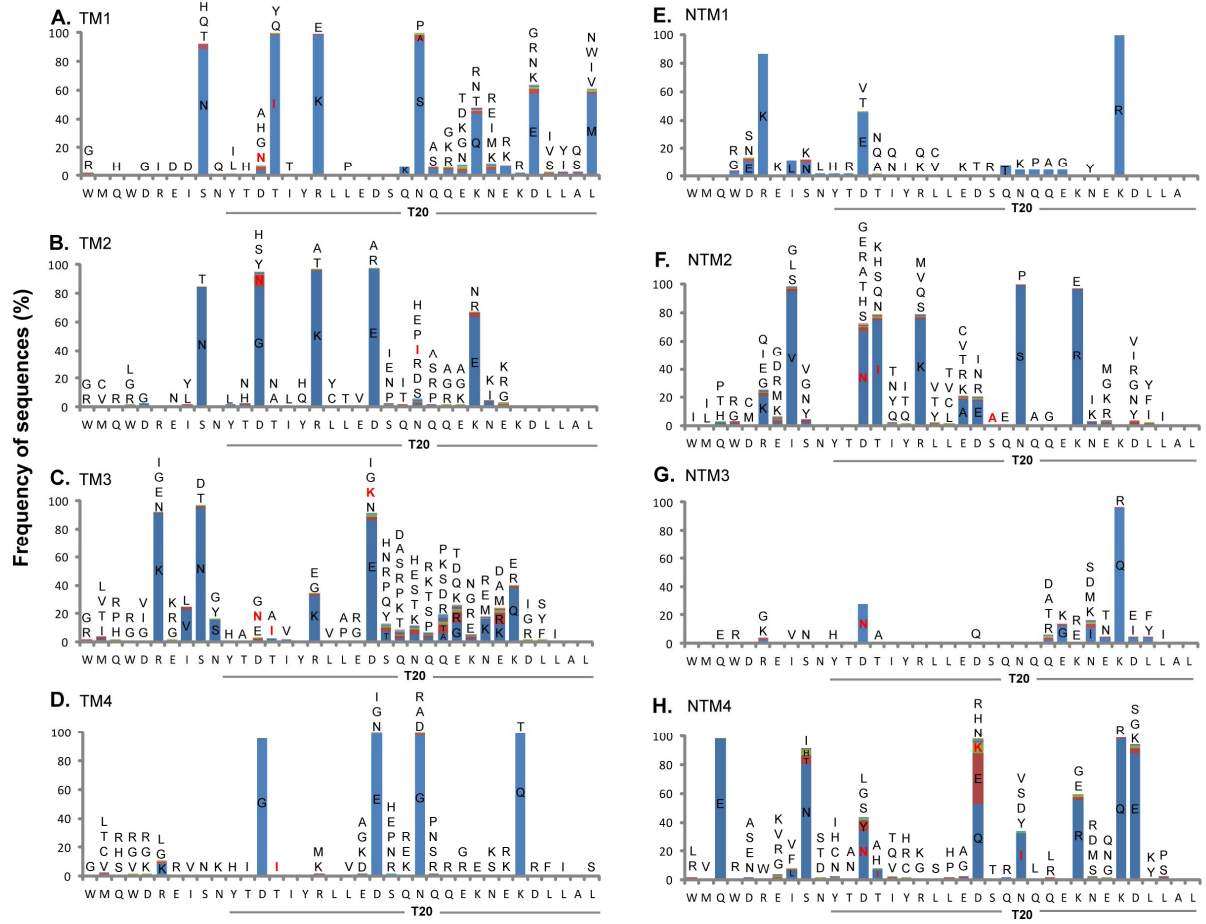

Fig. S3.

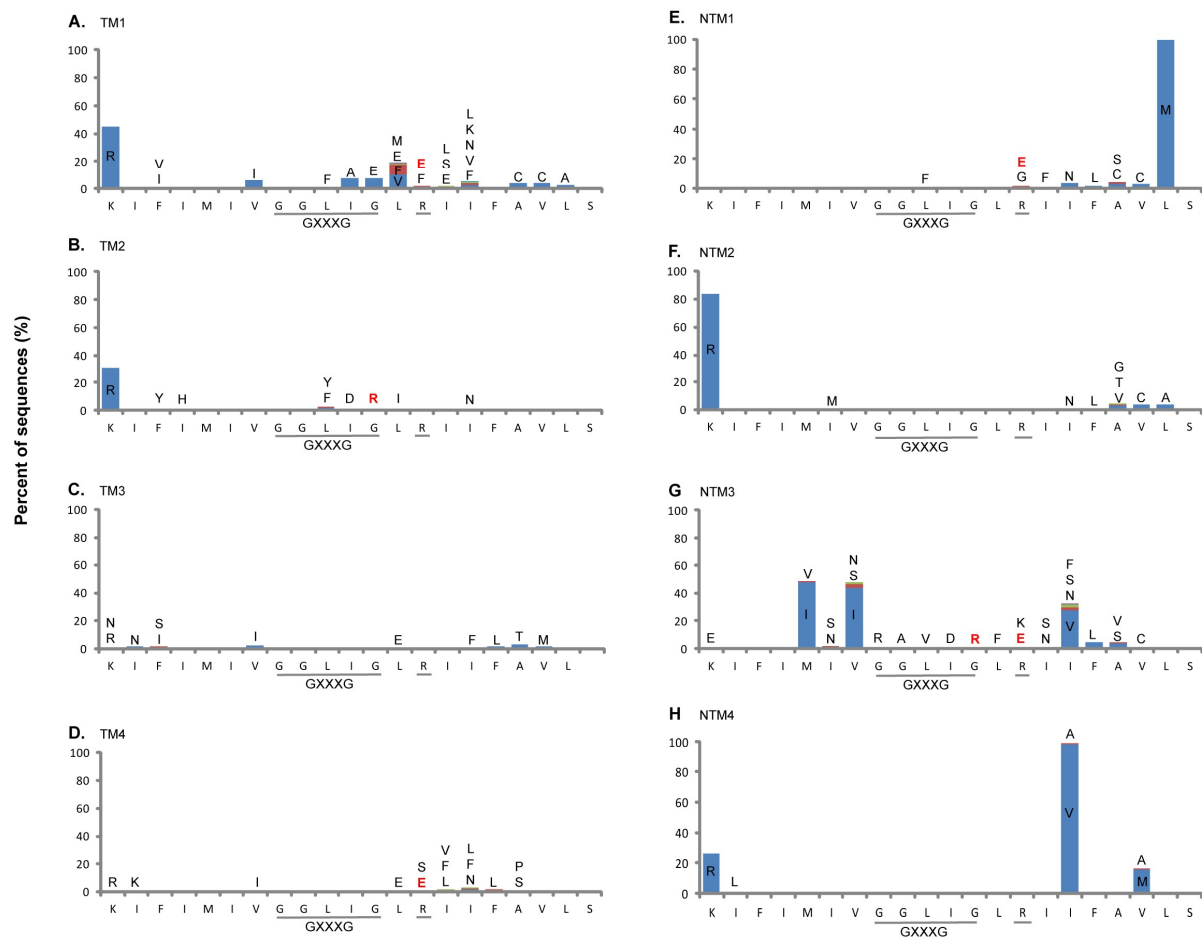

Supplement: JCPN-21-003_Supplementary file [file NIHMS1707957-supplement-JCPN-21-003_Supplementary_file.pdf]
